# CONTEXT AND TRUST: DIRECT CARE WORKER RELATIONSHIPS, CARE PRACTICES, FACILITATION, AND IMPLEMENTATION OF INNOVATIONS

**DOI:** 10.1093/geroni/igac059.1239

**Published:** 2022-12-20

**Authors:** Janelle Perez, Jing Wang, Anna Beeber, Ruth Anderson, Whitney Berta, Holly Lanham, Carole Estabrooks

**Affiliations:** University of North Carolina Chapel Hill, Chapel Hill, North Carolina, United States; University of North Carolina, Chapel Hill, North Carolina, United States; Johns Hopkins University, Baltimore, Maryland, United States; UNC Chapel Hill, Chapel Hill, North Carolina, United States; University of Toronto, Toronto, Ontario, Canada; University of Texas Health San Antonio, San Antonio, Texas, United States; University of Alberta, Edmonton, Alberta, Canada

## Abstract

This paper examines the relationships between Direct Care Workers (DCW) and other nursing home (NH) staff. Specifically, we examine how trust, and the context in which trust exists, influences care practices and implementation of innovations in NHs. This secondary analysis of interviews and field observations from three separate NHs in different Canadian provinces shows differing levels of trust that between HCAs and other staff. We also analyze the organizational context can mediate trust in these relationships. Specifically, we analyzed trust DCWs have at the interpersonal, institutional, organizational levels and what ways they act as facilitators of programs aimed at improving care practices health outcomes of residents in NHs. Using the Alberta Context Tool’s dimensions of organizational context as a framework, this research shows how trust in the relationships between DCWs and other LTC staff influences caregiving practices and the uptake of new innovations.

